# Comparison of the efficacy of combined budesonide and fexofenadine versus combined fluticasone propionate and fexofenadine on the expression of class-4 semaphorins and their receptors in the peripheral blood cells of patients with allergic rhinitis

**DOI:** 10.1016/j.heliyon.2023.e22924

**Published:** 2023-11-28

**Authors:** Gelayol Asadi, Parisa Feizollahi, Misagh Rajabinejad, Sara Falahi, Fatemeh Rezaei Varmaziar, Elham Faryadi, Ali Gorgin Karaji, Farhad Salari, Alireza Rezaiemanesh

**Affiliations:** a Student Research Committee, School of Medicine, Kermanshah University of Medical Sciences, Kermanshah, Iran; b Department of Immunology, School of Medicine, Kermanshah University of Medical Sciences, Kermanshah, Iran; c Department of Immunology, School of Medicine, Mazandaran University of Medical Sciences, Sari, Iran; d Student Research Committee, School of Medicine, Mazandaran University of Medical Sciences, Sari, Iran

**Keywords:** Allergic rhinitis, Class 4 semaphorins, Plexin, Fexofenadine, Fluticasone propionate, Budesonide

## Abstract

**Background:**

Allergic rhinitis (AR) is a common immunoglobulin (Ig) E-mediated disease. This study aimed to evaluate the gene expression levels of class 4 semaphorins and their receptors in AR patients before and after treatment with budesonide and fexofenadine (B/F) compared to fluticasone propionate and fexofenadine (FP/F).

**Methods:**

In this study, 29 AR patients (age 34.4 ± 1.2 years, 18 men and 11 women) were treated with B/F, and 24 AR patients (age 32.8 ± 1.9 years, 15 men and 9 women) were treated with FP/F for one month. Before and after treatment, peripheral blood samples were taken from patients. The expression levels of *SEMA4A*, *SEMA4C*, *SEMA4D*, *Plexin-B2*, and *Plexin-D1* genes were measured using the qPCR method. In addition, the serum levels of IgE were measured using an enzyme-linked immunosorbent assay (ELISA).

**Results:**

The expression levels of *SEMA4A* (*P* = 0.011), 4C (*P* = 0.017), *Plexin-B2* (*P* = 0.0005), and *Plexin-D1* (*P* = 0.008) remarkably increased in AR patients treated with B/F. Our results show a significant reduction in the gene expression levels of *SEMA4A* (*P* = 0.002), 4C (*P* = 0.014), 4D (*P* = 0.003), *Plexin-B2* (*P* = 0.033), and *Plexin-D1* (*P* = 0.035) after treatment with FP/F. The serum levels of IgE increased in FP/F treated group (*P* = 0.017) and conversely decreased in the treated group with B/F (*P* = 0.019). Moreover, the percentages of eosinophils were reduced in both FP/F and B/F groups (*P* = 0.015 and *P* = 0.0001, respectively).

**Conclusion:**

In conclusion, concomitant use of fexofenadine and fluticasone propionate reduced *SEMA4A*, 4C, 4D, *Plexin-B2*, and *Plexin-D1*, while the *SEMA4A*, 4C, *Plexin-B2*, and *Plexin-D1* gene expression levels were increased in the patient group treated with B/F.

## 1 Introduction

Allergic rhinitis (AR) is an immunoglobulin E (IgE) mediated type 1 hypersensitivity caused by inhaling a variety of environmental allergens [1], which affects more than 20 % of the worldwide population [2]. However, in some countries, this percentage was reported more than 50 % [[3], [4], [5]]. This chronic upper respiratory disease is characterized by typical symptoms, such as sneezing, nasal itching, rhinorrhea, and nasal congestion or obstruction, which implicated inflammatory responses to environmental allergens in the nasal mucosa [6]. In AR, the inflammatory circumstance is regulated through IgE-mediated responses. T helper 2 (Th2) cells immunological pattern, and activation of mast cells, leading to the release of inflammatory mediators and local recruitment of immune cells [[7], [8], [9]].

Semaphorins are a large family of secretary and membrane proteins that contribute to the nervous system as axon-conducting molecules with various functions, including the formation of the cerebral cortex, hippocampus, and spinal cord [10]. Semaphorins contain a homologous sequence of approximately 500 amino acids in the N-terminal extracellular domain, and they are divided into eight subclasses based on their terminal carboxyl domain (Sema domain). Class 4 semaphorins, including *SEMA4D*, *4C*, and *4B*, known as immune semaphorins, have received more attention due to their roles in the immune system following autoimmune disorders and allergic reactions [[11], [12], [13], [14]]. Immune semaphorins are expressed on the surface of various immune cells, including T cells, activated B cells, dendritic cells (DC), macrophages, neutrophils, and natural killer (NK) cells, contributing to regulating immune responses. For instance, *SEMA4C* plays a significant role in the polarization of B cells in the follicular helper T (Tfh) cell-dependent pathway [12,15].

Moreover, *SEMA4A* expressed on DCs can bind to the Tim-2 receptor on T cells and ultimately trigger antigen-specific immune responses [16]. *SEMA4D* also enhances CD40 signaling in B cells and DCs and implicates B cell isotype switching, affinity maturation, memory B cell generation, and DC activation [17,18]. It is also worth mentioning that plexins and neuropilins are the most characterized semaphorin receptors. *Plexin-B2*, expressed on B cells, macrophages, classical and plasmacytoid DCs, is a cell surface receptor for *SEMA4C*, *SEMA4D*, and *SEMA4A* [19,20]. *Plexin-D1* is also a receptor for *SEMA4A* and is expressed by thymocytes and activated B cells [21]. Given the evidence for the role of semaphorins in allergic diseases, evaluating the semaphorins mentioned above and their receptors may shed light on the mechanism underlying the pathogenesis of AR.

Antihistamines and intranasal corticosteroids (INCS) can be considered effective first-line therapy in patients with AR [22]. Fexofenadine, a second-generation antihistamine, reduces allergic symptoms such as sneezing, runny nose, itchy nose, and ocular symptoms in AR patients [23]. It also reduces allergic inflammatory responses mediated by mast cells, basophils, epithelial cells, eosinophils, and lymphocytes [24,25]. Fluticasone propionate and budesonide are intranasal corticosteroids that have been shown to ameliorate AR symptoms by reducing the number of nasal eosinophils, basophils, and neutrophils. Also, they inhibit T cell function and suppress the release of cytokines from mast cells [[26], [27], [28], [29]].

As we mentioned above, Class 4 semaphorins and their receptors are relevant in the context of allergic rhinitis, and changes in gene expression of semaphorins and their receptors may influence the pathogenesis of allergic rhinitis, which ultimately affect the clinical symptoms and outcomes of allergic rhinitis patients. Considering the indispensable contribution of semaphorins and their receptors in allergic diseases, this study was conducted to compare the efficacy of combined budesonide and fexofenadine versus combined fluticasone propionate and fexofenadine on the gene expression of *SEMA4A*, *SEMA4C*, *SEMA4D*, *Plexin-B2*, and *Plexin-D1* as well as evaluation of IgE levels and the percentages of eosinophil in the peripheral blood cells of patients with AR.

## 2 Materials and methods

### 2.1 Participants

Twenty-nine patients with AR (age 34.4 ± 1.2 years, 18 men and 11 women) who received a combination of fexofenadine and budesonide (F/B), and 24 patients with AR (age 32.8 ± 1.9 years, 15 men and 9 women) were treated with the combination of fexofenadine and fluticasone propionate (FP/F) were included in this study. The demographic data of the study participants are listed in Table 1. An allergist diagnosed and selected patients with AR according to a comprehensive history and physical examination. (29). For all patients, under the supervision of the physician, corticosteroids, including Prednisolone, Dexamethasone, Beclomethasone, Mometasone, etc., were discontinued two months before starting new treatments (B/F or FP/F). Intranasal fluticasone propionate and budesonide were administered in two puffs daily and fexofenadine (one pill daily, 60 mg). Patients with other antiallergic medicines and other inflammatory diseases such as pharyngitis, laryngitis, autoimmune diseases, cancer, infectious diseases, and pregnant women were excluded from the study. Informed consent forms were obtained from all patients. This study was performed with approval from the Ethics committee of the Kermanshah University of Medical Sciences (IR.KUMS.REC.1399.275), and it was approved with two clinical trial registration numbers: IRCT2017050233622N2 and IRCT2017042433622N2 in the Iranian Registry of Clinical Trials (IRCT).

**Table 1:** Characteristics of patients with allergic rhinitis were included in the study.

Groups	Patients (number)	Age (yrs) (Mean ± SD)	Sex (Male/Female)	Duration of AR (yrs)
**B/F**	29	32.8 ± 1.9	18.11	8 ± 1.7
**FP/F**	24	34.4 ± 1.2	15.9	5.8 ± 0.8

AR; Allergic rhinitis, FP/F; fluticasone propionate and fexofenadine, B/F; budesonide and fexofenadine, SD; Standard Deviation, yrs; years.

### 2.2 Study design

Peripheral blood samples were collected from patients before and after one-month treatment and transferred into the tube containing EDTA to conduct further experiments. A blood smear was also prepared to count the percentage of eosinophil cells.

### 2.3 Serum levels of IgE

Serum IgE levels were measured using the enzyme-linked immunosorbent assay (ELISA) method before and after treatment in AR patients according to the manufacturer's instructions (IgE EIA Kit, Pad-tan Elm, Iran).

### 2.4 RNA extraction, cDNA synthesis, and real-time PCR

RNA content was extracted from EDTA-containing blood samples according to the manufacturer's protocol (Yekta Tajhiz Azma, Iran). The extracted RNA was converted into complementary DNA (cDNA), based on the manufacturer's instructions (Yekta Tajhiz Azma, Iran), using a thermocycler (Bio-rad Thermal Cycler C1000 Touch system, Germany). The expression levels of the target genes were determined using a real-time PCR system (LightCycler 96 system (Roche Molecular Biochemicals, Mannheim, Germany) and SYBR Green PCR master (Ampliqon Inc., Odense, Denmark). 18S rRNA was used to normalize mRNA expression levels of the target genes, including *SEMA4A*, *SEMA4C*, *SEMA4D*, *Plexin-B2*, and *Plexin-D1*. Details of the designed primers are shown in Table 2. Relative changes in transcriptional levels of mRNAs were calculated using the 2^−ΔΔCt^ formula (*pfaffl* method).

**Table 2:** Primers sequences are used in real-time PCR quantification.

Gene name	Sequences (5′–3′)
*SEMA4A*	F: 5ʹ-GCTTGTACCTTCATTGAACTTC-3ʹ
	R: 5ʹ-CATAGTACCAGAATAGAGCATCC-3ʹ
*SEMA4C*	F: 5ʹ-CTGCGCGCAATAGGACAG-3ʹ
	R: 5ʹ-CAGCCAGACAGCCCAGTG-3ʹ
*SEMA4D*	F: 5ʹ-GAAGCAGCATGAGGTGTATT-3ʹ
	R: 5ʹ-GGATGTTAAGTTCAGGTGGTC-3ʹ
*PLXNB2*	F: 5ʹ-CTACTGCTACTGGAGGAAGAGC-3ʹ
	R: 5ʹ-ATTCCTTCTTGCAGCGGTCC-3ʹ
*PLXND1*	F: 5ʹ-AATGGGCGGAACATCGTCAAG-3ʹ
	R: 5ʹ-CGAGACTGGTTGGAAACACAG-3ʹ
18s rRNA	F: 5ʹ-GTAACCCGTTGAACCCCATT-3ʹ
	R: 5ʹ-CCATCCAATCGGTAGTAGCG-3ʹ

### 2.5 Statistical analysis

Data were statistically analyzed using SPSS software version 16 (SPSS, Chicago, IL, USA) and GraphPad Prism version 8 (GraphPad, Software, La Jolla, California, USA). The Kolmogorov–Smirnov test analyzed the normality of data distribution. The nonparametric Wilcoxon signed-rank test was used to compare data. Data were expressed as means ± standard deviation (SD), and a *P* value less than 0.05 was considered a statistically significant level.

## 3 Results

### 3.1 The mRNA expression levels of *SEMA4A*, *4C*, *4D* and their receptors in the patients treated with B/F

**Fig. 1:**
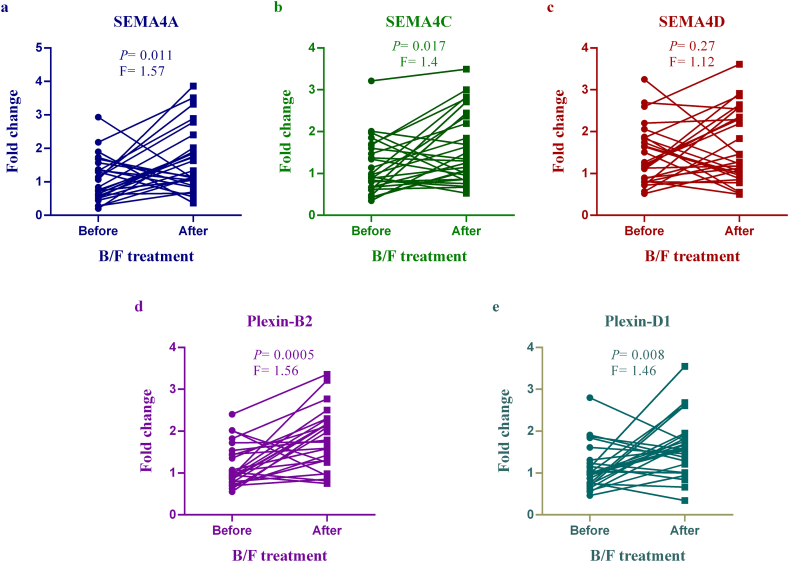
mRNA expression of class-4 semaphorins and their receptors in the B/F group. Real-time PCR was used to detect the transcriptional level of *SEMA4A* (a), *SEMA4C* (b), *SEMA4D* (c), *Plexin-B2* (d), and *Plexin-D1* (e) in the peripheral blood samples obtained from AR patients before and after treatment with B/F. (AR; Allergic rhinitis, B/F; budesonide and fexofenadine, F; Fold change).

As shown in Fig. 1 (a-c), the expression levels of *SEMA4A* and *SEMA4C* genes increased significantly in patients treated with B/F compared to before treatment (P = 0.011 and P = 0.017, respectively). In contrast, the expression levels of *SEMA4D* had no remarkable difference (P = 0.27). We also measured *Plexin-B2* and *Plexin-D1* expression levels before and after treatment. The provided data depicts significantly elevated expression levels of *Plexin-B2* (P = 0.0005) and *Plexin-D1* (P = 0.008) after one month of treatment (Fig. 1 d and e).

### *3.2* The mRNA expression levels of *SEMA4A*, 4C, 4D and their receptors in the patients treated with FP/F

**Fig. 2:**
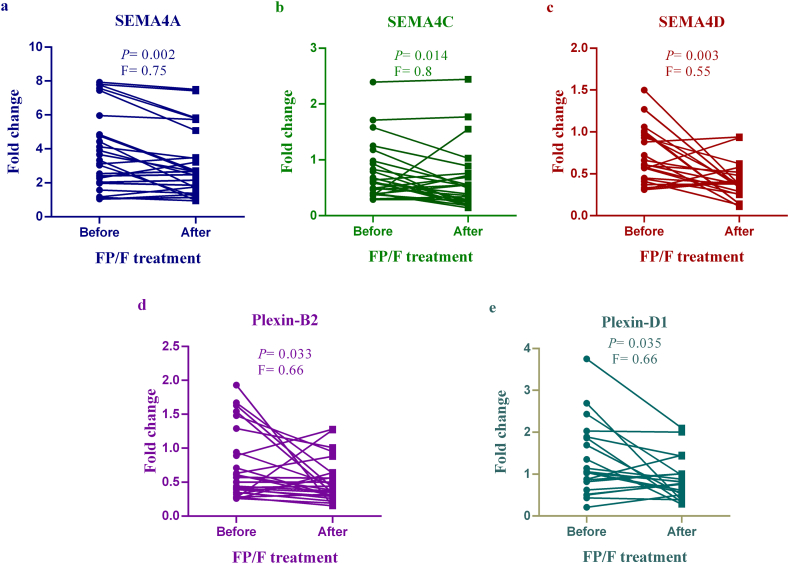
mRNA expression of Class-4 semaphorins and their receptors in the FP/F group. Real-time PCR was used to detect the transcriptional level of *SEMA4A* (a), *SEMA4C* (b), *SEMA4D* (c), *Plexin-B2* (d), and *Plexin-D1* (e) in the peripheral blood samples obtained from AR patients before and after treatment with FP/F. (AR; Allergic rhinitis, FP/F; fluticasone propionate and fexofenadine, F; Fold change).

As shown in Fig. 2 (a-e), our provided data showed that *SEMA4A* (P = 0.002), *SEMA4C* (P = 0.014), *SEMA4D* (P = 0.003), *Plexin-B2* (P = 0.033) and *Plexin-D1* (P = 0.035) mRNA expression levels were remarkably decreased in FP/F treated patients compared to before treatment.

### 3.3 The effect of B/F and FP/F on the serum levels of IgE and percentage of eosinophils

**Fig. 3:**
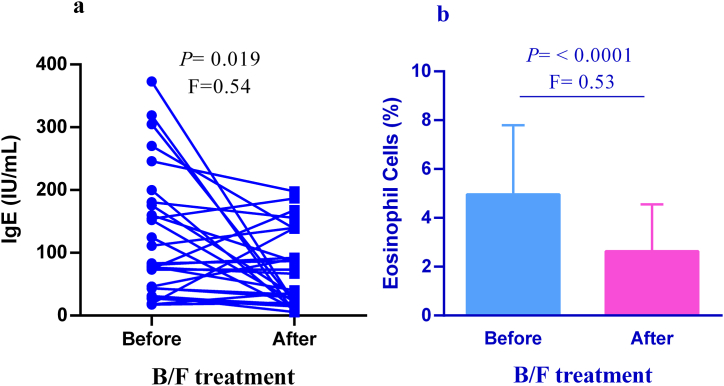
The serum IgE levels (a) and percentage of eosinophil cells (b) in AR patients before and after treatment with B/F (AR; Allergic rhinitis, B/F; budesonide and fexofenadine, F; Fold change).

**Fig. 4:**
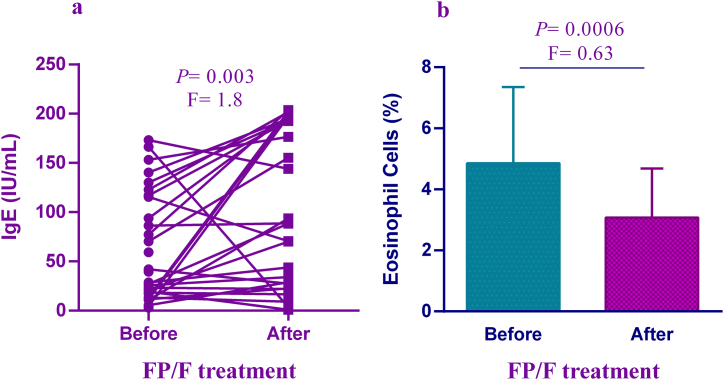
The serum IgE levels (a) and percentage of eosinophil cells (b) in AR patients before and after treatment with FP/F (AR; Allergic rhinitis, FP/F; fluticasone propionate and fexofenadine, F; Fold change).

The IgE serum levels were significantly reduced in AR patients treated with B/F, (P = 0.019; Fig. 3a). As such, the percentage of eosinophils was considerably decreased in patients treated with B/F compared to before treatments (P < 0.0001; Fig. 3b). In the following, the given data indicated a significant elevation in the serum levels of IgE in AR patients treated with FP/F compared to before treatment (P = 0.003; Fig. 4a), while the percentage of eosinophils was significantly decreased in the mentioned group after treatments (P = 0.0006; Fig. 4b).

### 3.4 Effect of treatment on the clinical symptoms

The allergic reaction changes were analyzed before and after treatment to determine whether B/F and FP/F could suppress the allergic symptoms in AR. Our results showed an overall significant reduction in all clinical symptoms of patients, including sneezing, airway obstruction, nasal and eye itching, and eye and nose watery after treatment with the B/F and FP/F compared to before treatment (Supplementary data).

## 4 Discussion

The pathogenesis of Allergic rhinitis is attributed to the polarization of the Th2 cells and IgE-stimulated mast cells and eosinophils [30]. Antihistamines and corticosteroids are routinely prescribed together in AR patients, and studies verified that combination therapy had better outcomes than monotherapy in patients with AR [[31], [32], [33]]. Although many studies have been conducted on the investigation of therapeutic benefits of intranasal corticosteroids and oral antihistamines in the treatment of AR and their mechanism of action, the effect of fexofenadine, fluticasone propionate, and budesonide on the immune cells and inflammatory mediators involved in the development of AR has been less known.

In the present study, we assessed the gene expression of semaphorin *4A*, *4C*, and *4D* as well as their receptors (*Plexin-B2* and *D1*) before and after one month of treatment with combined budesonide and fexofenadine (B/F) and combined fluticasone propionate and fexofenadine (FP/F). Our results showed that the expression levels of *SEMA4A*, *SEMA4C*, *Plexin-D1*, and *Plexin-B2* in the B/F treated patients group were significantly increased, while the *SEMA4D* had no considerable differences. Conversely, the expression levels of the mentioned genes remarkably decreased after one month of FP/F treatment to before treatment. To note, Semaphorins, particularly class 4, contributed to the immune system function, and perturbations of semaphorins signaling have been associated with immune system-related diseases, including rheumatoid arthritis, multiple sclerosis, systemic lupus erythematosus, and allergic diseases [34,35]. A study by Elizabeth et al. demonstrated the *SEMA4A* and *SEMA4C* expression has increased in the lung with allergen-induced inflammation [36]. Likewise, it has been reported that the levels of interleukin (IL)-5, IL-6, IL-13, IL-17A, and transforming growth factor-beta 1 (TGF-β1) reduced in allergen-treated Sema4D^−/−^ mice relative to wild type mice [37]. Moreover, inhibition of *SEMA4A*-*Plexin-D1* reduced lung fibrosis mediated by AKT (protein kinase B) signaling [38]. Interestingly, Another survey declared that *SEMA4C* could be a key player in local allergic inflammation [39]. It has also been shown that *SEMA4A*-*Plexin-D1* contributed to the elevated IL-4 and IL-17 in patients with systemic sclerosis (SSc), and the same manner can be seen for other T CD4^+^ mediate diseases, including asthma, rheumatoid arthritis (RA), and multiple sclerosis (MS) [[40], [41], [42]].

In concert with the inflammatory role of immune semaphorins, *Plexin-D1*, and *B2* in allergic diseases and concerning the effect of FP/F on the reducing expression of mentioned genes, FP/F may play a role in regulating *SEMA4C*, *4D*, *4A* and their receptors, and subsequently, modulate adverse allergic responses in AR patients compared to B/F. Although B/F leads to improvement in the clinical symptoms of AR patients, it increases the gene expression of *SEMA4A* and 4C, suggesting B/F probably targets another signaling pathway in ameliorating the clinical symptoms of AR patients. It is also noteworthy that the signaling pathway mechanisms of fexofenadine, fluticasone propionate, and budesonide in AR were not cleared and needed to be further investigated.

In the following, we assessed the serum levels of IgE and eosinophil percentages in both treated patient groups with B/F and FP/F. Our data showed elevated IgE levels and a reduction in the percentage of eosinophils in patients undergoing FP/F. While in B/F treated patients, both of them decreased.

Previous studies have demonstrated controversial results regarding the impact of antihistamines and corticosteroids on IgE levels. The research performed by Arun et al. pointed out that fexofenadine had no significant effect on the serum IgE levels in patients with AR [43]. Intriguingly, it was also shown that glucocorticoid treatment increased IgE concentrations in patients with asthma [44,45]. One of the reasons for the increase of IgE levels following treatment with corticosteroids is their impact on the stimulation of IgE production through IL-4 signaling, which has been observed in B cells treated with hydrocortisone [46]. In contrast, another study revealed that fluticasone propionate had no significant effects on the boosts of systemic allergen-specific IgE [47]. Collectively, these data can justify the elevated IgE levels following treatment with FP/F. However, a consensus on the effect of corticosteroids on IgE has not been attained.

Finally, some potential limitations to our study should be reminded. The sample size was relatively small, and we could not assess the semaphorin protein levels.

Regarding the ameliorating symptoms of AR in patients, it can be concluded that both drug combinations used in treating rhinitis are effective. Still, their effectiveness pathway is different, and further investigation needs to clarify their mechanisms.

## 5 Conclusion

To sum up, concomitant use of fexofenadine and fluticasone propionate reduced *SEMA4A*, 4C, 4D, *Plexin-B2*, and *Plexin-D1* expression levels, while the *SEMA4A*, 4C, *Plexin-B2*, and *Plexin-D1* gene expression were increased in the patients group treated with B/F.

## Ethical statement

This study was performed with approval from the Ethics committee of the 10.13039/501100005317Kermanshah University of Medical Sciences (IR.KUMS.REC.1399.275), and it was approved with two clinical trial registration numbers: IRCT2017050233622N2 and IRCT2017042433622N2 in the Iranian Registry of Clinical Trials (IRCT). All research was performed in accordance with relevant guidelines and regulations of 10.13039/501100005317Kermanshah University of Medical Sciences.

## Consent for publication

Written informed consent was obtained from all participating individuals.

## Funding

This research has been supported by grants from 10.13039/501100005317Kermanshah University of Medical Sciences (KUMS); Grant No. 990367.

## Authors' contributions

G.A. and F.R.V. conceived the study and wrote the manuscript. P.F. and S.F. performed the laboratory experiments. A.G.K., M.R. and E.F. provided methodological support in the study design. A.R. and F.S. critically revised the manuscript and provided the final approval. All authors read and approved the final manuscript.

## Data availability statement

Data will be made available on request.

## CRediT authorship contribution statement

**Gelayol Asadi:** Writing – original draft, Methodology. **Parisa Feizollahi:** Methodology, Data curation. **Misagh Rajabinejad:** Writing – original draft, Formal analysis. **Sara Falahi:** Methodology, Data curation. **Fatemeh Rezaei Varmaziar:** Methodology, Investigation. **Elham Faryadi:** Writing – original draft. **Ali Gorgin Karaji:** Resources, Conceptualization. **Farhad Salari:** Writing – review & editing, Validation. **Alireza Rezaiemanesh:** Writing – review & editing, Supervision, Resources, Methodology, Funding acquisition, Formal analysis, Conceptualization.

## Declaration of competing interest

The authors declare that they have no known competing financial interests or personal relationships that could have appeared to influence the work reported in this paper.
