# Supplementary material for: Comparison of the efficacy of combined budesonide and fexofenadine versus combined fluticasone propionate and fexofenadine on the expression of class-4 semaphorins and their receptors in the peripheral blood cells of patients with allergic rhinitis
Source: Heliyon. 2023 Nov 28;10(1):e22924. doi: 10.1016/j.heliyon.2023.e22924 (PMC10750067; doi:10.1016/j.heliyon.2023.e22924)
Supplement: Multimedia component 1 [file mmc1.docx]

**Supplementary table 1.** Clinical symptoms of patients with allergic rhinitis before and after treatments.

| Clinical symptoms | Sneezing  (Mean ± SD) | Rhinorrhea  (Mean ± SD) | Airway obstruction  (Mean ± SD) | Itching nose  (Mean ± SD) | Itching eye  (Mean ± SD) | Watery eye  (Mean ± SD) |
| --- | --- | --- | --- | --- | --- | --- |
| Before B/F treatment | 4.03± 0.30 | 3.96± 0.34 | 3.62± 0.26 | 3.28± 0.42 | 1.96± 0.27 | 1.93± 0.31 |
| After B/F treatment | 0.72± 0.18 | 0.69± 0.17 | 0.62± 0.24 | 0.55± 0.20 | 0.41± 0.18 | 0.38± 0.12 |
| *P* value | **<0.0001** | **<0.0001** | **<0.0001** | **<0.0001** | **<0.0001** | **<0.0001** |
| Before FP/F treatment | 5.2 ± 0/28 | 5.32 ± 0.26 | 4.16 ± 0.40 | 3.28 ± 0/46 | 2.84 ± 0.49 | 2.96 ± 0.43 |
| After FP/F treatment | 1 ± 0/26 | 0.88 ± 0.33 | 0.48 ± 0.19 | 0.56 ± 0/17 | 0.64 ± 0.26 | 0.36 ± 0.22 |
| *P* value | **<0.0001** | **<0.0001** | **<0.0001** | **<0.0001** | **<0.0001** | **<0.0001** |

FP/F; fluticasone propionate and fexofenadine, B/F; budesonide and fexofenadine; SD; Standard Deviation.
